# AI Chatbots for Psychological Health for Health Professionals: Scoping Review

**DOI:** 10.2196/67682

**Published:** 2025-03-19

**Authors:** Gumhee Baek, Chiyoung Cha, Jin-Hui Han

**Affiliations:** 1 College of Nursing, Ewha Womans University, 52 Ewhayeodae-gil, Daehyun-dong, Seodaemun-gu, Seoul, 03760, Republic of Korea, 82 1035065701; 2 Graduate Program in System Health Science and Engineering, Ewha Womans University, Seoul, Republic of Korea; 3 College of Nursing, Ewha Research Institute of Nursing Science, Ewha Womans University, Seoul, Republic of Korea

**Keywords:** artificial intelligence, AI chatbot, psychological health, health professionals, burnout, scoping review

## Abstract

**Background:**

Health professionals face significant psychological burdens including burnout, anxiety, and depression. These can negatively impact their well-being and patient care. Traditional psychological health interventions often encounter limitations such as a lack of accessibility and privacy. Artificial intelligence (AI) chatbots are being explored as potential solutions to these challenges, offering available and immediate support. Therefore, it is necessary to systematically evaluate the characteristics and effectiveness of AI chatbots designed specifically for health professionals.

**Objective:**

This scoping review aims to evaluate the existing literature on the use of AI chatbots for psychological health support among health professionals.

**Methods:**

Following Arksey and O’Malley’s framework, a comprehensive literature search was conducted across eight databases, covering studies published before 2024, including backward and forward citation tracking and manual searching from the included studies. Studies were screened for relevance based on inclusion and exclusion criteria, among 2465 studies retrieved, 10 studies met the criteria for review.

**Results:**

Among the 10 studies, six chatbots were delivered via mobile platforms, and four via web-based platforms, all enabling one-on-one interactions. Natural language processing algorithms were used in six studies and cognitive behavioral therapy techniques were applied to psychological health in four studies. Usability was evaluated in six studies through participant feedback and engagement metrics. Improvements in anxiety, depression, and burnout were observed in four studies, although one reported an increase in depressive symptoms.

**Conclusions:**

AI chatbots show potential tools to support the psychological health of health professionals by offering personalized and accessible interventions. Nonetheless, further research is required to establish standardized protocols and validate the effectiveness of these interventions. Future studies should focus on refining chatbot designs and assessing their impact on diverse health professionals.

## Introduction

Health professionals are on the front line of patient care, enduring enormous responsibilities and constant pressure. Their work often occurs in an environment in which they must make life-and-death decisions, making them particularly vulnerable to psychological burnout [1]. According to recent studies, more than 50% of health professionals worldwide experience symptoms of burnout, such as fatigue, cynicism, and decreased effectiveness, leading to low job satisfaction [2], high turnover [3], and low-quality patient care [4]. This phenomenon not only causes an individual crisis but also has broader implications for health care systems [2,4]. Since the COVID-19 pandemic, psychological health issues such as anxiety, depression, and burnout among health professionals have become more prevalent because of long working hours, high-stress working environments, and exposure to traumatic events [5]. These issues directly affect health professionals’ well-being and work performance and can ultimately negatively impact the quality of health care [6]. Therefore, supporting the psychological health of health professionals and preventing burnout are urgent challenges.

While traditional face-to-face counseling has been used to prevent psychological illness among health professionals, this method presents various limitations including temporal and spatial constraints, limited availability of mental health professionals, accessibility barriers, and anonymity concerns [7-9]. Health professionals face significant challenges in accessing psychological care owing to demanding work schedules and high work intensity [10]. Additionally, they may feel psychologically burdened when seeking help in situations in which anonymity is not guaranteed [8]. To address these challenges, artificial intelligence (AI) chatbots have emerged as a potential solution, offering a new way to deliver psychological health interventions that can compensate for the limitations of traditional methods [7,8]. AI chatbots have the potential to be accessible anytime and anywhere while maintaining user anonymity, which may provide an alternative approach to psychological health support [7].

Recently, rapid advances in AI and natural language processing (NLP) have opened new possibilities for psychological health interventions, with AI chatbots gaining particular attention in the field of psychological health. Studies have demonstrated the potential of AI chatbots—AI-driven conversational agents—in providing mental health support [11] while emphasizing the importance of their safe and explainable implementation [12]. AI chatbots analyze users’ conversation histories and data to provide advice and support tailored to their specific needs. This can help health professionals better manage psychological health issues, such as burnout, and enable timely interventions tailored to individual situations [13]. Previous research has shown that AI chatbots can improve coping skills through natural conversations with users [7], enhance the user experience, facilitate effective intervention delivery [13,14], and provide interventions tailored to specific needs [13,14]. This conversational approach enhances the effectiveness of interventions by fostering positive user engagement, as people are more likely to empathize with and respond to tools that feel engaging and relatable [6,7,14].

Despite the potential of AI chatbots for enhancing psychological health, there remains a lack of research on their effectiveness for health professionals. Much of the existing research has focused on using AI chatbots in patient care, whereas relatively little research exists on the use of AI chatbots to support health professionals. This research gap highlights the need to systematically investigate the characteristics and effectiveness of AI chatbots that target health professionals. Therefore, this study aimed to fill this gap and analyze the characteristics and effectiveness of AI chatbot interventions targeting health professionals to contribute to future research and practice.

## Methods

### Study Design

We conducted a scoping review to identify the available evidence regarding AI chatbot interventions for health professionals’ psychological health. A scoping review maps the relevant literature in the field and is useful for identifying gaps in knowledge [15]. This review was conducted in accordance with the scoping review framework of Arksey and O’Malley. The study adopted the following five steps: (1) identifying the research question; (2) identifying relevant studies; (3) study selection; (4) charting the data; and (5) collating, summarizing, and reporting the results. The five stages provided a structured approach to assist in understanding the current evidence by examining the literature. There was no rating of quality performed on the included studies, as scoping studies do not require a quality evaluation [16]. Therefore, a scoping review is suitable for providing a broader perspective on the current research and mapping the relevant literature on AI chatbot interventions for health professionals’ psychological health.

### Identifying the Research Question and Relevant Studies

The research question was, “What is the status of the current body of knowledge regarding AI chatbots for the psychological health of health professionals?” According to Arksey and O’Malley, the search strategy involves identifying relevant studies, including published and unpublished works, with an emphasis on being as comprehensive as possible [15]. A literature search was performed between May and August 2024 from the following eight databases: seven global search engines in English (Cochrane Library, CINAHL, Embase, ProQuest Dissertations & Theses Global, PsyclNFO, PubMed, and Web of Science) and one Korean search engine in Korean (Research Information Sharing Service). Various combinations of search terms (Textbox 1) were used to either broaden or narrow the search, depending on the results in a specific database. The reference lists of the retrieved studies were searched manually. Finally, gray literature, relevant letters, and editorials were searched.

Textbox 1. **Query box**

medical team OR medical staff* OR healthcare worker* OR healthcare provider* OR health personnel* OR health professional* OR doctor* OR surgeon* OR physician* OR nurse* OR midwife* OR Licensed Practical Nurse* OR licensed vocational nurse* OR physician assistant OR nurse practitioner OR clinical nurse specialist* OR nurse clinician* OR registered nurse* OR resident* OR therapist* OR pharmacist* OR nurse assistant* OR nurse aid* AND burnout OR burn-out* OR depress* OR depression OR stress* OR exhaust* OR depersonalization OR personal accomplishment OR anxiety* AND chatbot* OR artificial intelligence OR conversational agent* OR dialogue system* OR machine learning OR natural language process*

### Study Selection

#### Overview

Studies published before May 2024 were selected for this review. The studies retrieved from the literature search were reviewed by three researchers using the following inclusion and exclusion criteria.

#### Inclusion Criteria

(1) The purposes of the studies included the development or implementation of AI chatbot interventions. (2) Participants of the studies included licensed health professionals such as physicians or surgeons, dentists, medical doctors, registered nurses, licensed practice or vocational nurses, pharmacists, and allied health professionals such as respiratory and physical therapists. Studies that included nonhealth professionals in addition to health professionals as part of their participants were included if the main purpose of the AI chatbot was to alleviate the psychological distress of health professionals. (3) The intention of the AI chatbots was to improve the psychological health of health professionals, focusing on outcomes such as stress, burnout, depression, and anxiety. (4) The studies were written in English or Korean.

#### Exclusion Criteria

Studies in which we could not retrieve the full text were excluded.

**Figure 1.**
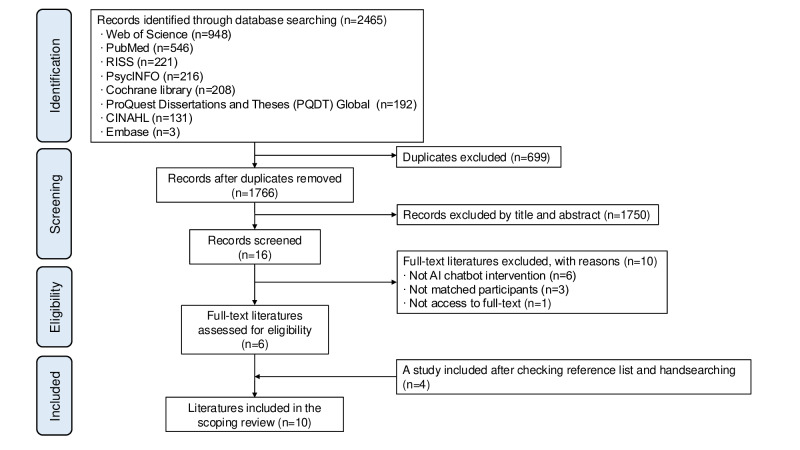
Flowchart. AI: artificial intelligence.

A total of 2465 studies were retrieved from the initial database search (Figure 1). After removing duplicates, 1766 studies remained. The titles and abstracts were reviewed, and 1750 studies were excluded, leaving 16 studies for a full-text review. Six studies completely met the inclusion criteria after full-text review. Then, by reviewing the reference lists and manually searching, five additional studies were obtained, of which four studies met the criteria for the scoping review. Finally, 10 studies were included in this scoping review.

Throughout the review process, two researchers (GB and JH) took the lead in data extraction, and all researchers reviewed the content. Any uncertainties and disagreements were resolved through weekly meetings. The literature review software Covidence (Veritas Health Innovation Ltd), a study-managing tool for collaborating researchers [17], was used to manage the retrieved studies and to screen for redundant studies.

### Charting the Data

The data are presented in tables, summarized, and synthesized qualitatively according to the purpose and research questions of the review.

### Collating, Summarizing, and Reporting the Results

The final stage of a scoping review involves collating, summarizing, and reporting the results [15]. Data extraction was performed by two researchers (GB and JH) and checked for accuracy by all the researchers. Uncertainties and disagreements were resolved through discussions. The data were extracted using a common data extraction format.

## Results

### General Characteristics

Table 1 presents the characteristics of the analyzed studies. Ten studies published between 2018 and 2024 were retrieved. The studies were published worldwide: in Canada [7,18], Japan [19], Malawi [20], Netherlands [21], Singapore [13,22], Spain [23], and the United States [24,25]. Four studies examined diverse health professionals, including doctors, nurses, nurse assistants, clinical officers, pharmacists, lab technicians, and other allied health professionals [20,21,23,26]. Two studies specifically targeted doctors and nursing staff [13,27] such as nurse practitioners, registered nurses, licensed practical nurses, and nursing assistants, while one study exclusively covered chatbots for nurses [19]. Three studies identified their participants as health professionals, without specifying the specific types [7,18,22]. Of the ten studies, eight studies focused solely on health professionals [13,19-23,26,27]. Two studies included both health professionals and their families [7,18].

The study designs were diverse. Of the 10 studies, three were development studies [18,23,27], one was a design verification study [26], one was a pilot study [22], one was a randomized controlled trial [20], one was a mixed-method feasibility study [21], one was an intervention study [19], one was a cross-sectional study protocol study [7], and one was a qualitative study [13]. Three studies evaluated the development and feasibility of AI chatbots for psychological health support [23,26,27].

**Table 1.** Study characteristics.

Author (year)/ country	Study design	Participants	Source of participants	Sample size (M:F^a^ ratio)	Age (years), mean (SD)	Publication type
Anmella et al [27](2023)/Spain	Development study	Primary care and health care professionals; medical doctors (GPs^b^ and specialists from diverse disciplines), nurses, and nurse assistants	GP referrals to PCMHSP^c^ Hospital Clínic de Barcelona health professionals	Simulation study: 17 (24:76) Feasibility and effectiveness study: 34 (24:76)	Simulation study: 36.5 (9.7) Feasibility and effectiveness study: 35.3 (10.12)	Journal study
Chaudhry and Islam [26](2021)/United States	Design verification study	Health professionals in their workplaces during the COVID-19 pandemic; RNs^d^, NPs^e^, LPN^f^, CNAs^g^, PAs^h^, RT^i^, PTs^j^, students or intern nurses	Local medical services departments and health professionals’ mailing lists	22 (9.1:90.9)	34.98 (8.55)	Journal study
Chang et al [22] (2024)/Singapore	Pilot exercise (service evaluation study)	Health professionals (types unspecified)	National tertiary health care cluster	527 (N/A)^k^	N/A	Journal study
Jackson-Triche et al [23] (2023)/United States	Development and usability study	Health professionals including faculty, staff, and trainees across various disciplines within the health care system	UCSF^l^	Over 26,000 (N/A)	N/A	Journal study
Kleinau et al [20] (2024)/Malawi	Randomized controlled trial	Health professionals (doctors, nurses, clinical officers, medical assistants, pharmacists, lab technicians, physiotherapy technicians)	Public and private health care facilities within Blantyre and Lilongwe districts	1584 (N/A) Completed: 511 (E=296, C=215)	N/A	Journal study
Kroon [21](2018)/Netherlands	Mixed method feasibility study (one group pre-posttest design)	Health professionals (radiation expert, social worker, nurse in training, training officer, pedagogue, health professional disability care HRD^m^ adviser	Email using personal and iThrive connections.	12 (8.3:91.7) (interview participant=9)	Half of the participants were aged 18 to 25 years	Master’s thesis
Matsumoto et al [19](2021)/Japan	Intervention study	Nurses	Two hospitals in Tokyo	70 (N/A)	N/A	Journal study
Noble et al [7](2022)/Canada	Cross-sectional study protocol	Health professionals and their families	The Canadian provinces of Alberta and Nova Scotia	2200 (N/A)	N/A	Journal study
Yoon et al [13] (2021)/Singapore	Qualitative study	Frontline health professionals (doctors and nurses)	Singapore general hospital and community care facilities (large scale institutional isolation units)	42 (24:76)	29.6 (3.9)	Journal study
Zamani [18](2022)/Canada	Development study	Health professionals and their families	The Canadian provinces of Alberta and Nova Scotia	N/A	N/A	Master’s thesis

a M:F: male:female.

b GP: general practitioner.

c PCMHSP: Primary Care Mental Health Support Program.

d RN: registered nurse.

e NP: nurse practitioner.

f LPN: licensed practical nurse.

g CNA: certified nurse assistant.

h PA: physician assistant.

i RT: respiratory therapist.

j PT: physical therapist.

k N/A: not available.

l UCSF: University of California, San Francisco

m HRD: human resource development.

### AI Chatbot Characteristics

Table 2 shows the characteristics of the AI chatbots developed or implemented in the retrieved studies. The types of AI chatbot algorithms were diverse; NLP-based AI was the most commonly used algorithm, appearing in six studies [7,13,18,19,21,23]; rule-based AI, an algorithmic method that does not learn from data or create rules on its own, was used in two studies [20,22]; and learning-based AI that learns from data was adopted in two studies [26,27]. AI chatbots were delivered through mobile apps in six studies [13,19,20,22,26,27], and web-based platforms via web links in four studies [7,18,21,23]. The interaction type in all studies was 1:1 interaction: Among them, one study included 1:1 interaction alongside group chat functionality [26], and another study included 1:1 interaction with optional human coach support [22]. In seven studies, the chatbot actively initiated the conversation [7,13,18-20,23,27], while in three studies, the conversation was passively initiated [21,22,26].

Participant completion of the AI chatbot interventions was reported in four studies [20,21,23,27]; among these, three studies provided the percentage of participants who completed the intervention: 14.3% [21], 67.7% [20], and 73% [27] while one study reported the employee access rate of the intervention as 10.88% [27]. Of the 10 studies, six evaluated the AI chatbot’s usability [13,20,22,23,26,27]. Four studies relied on participant feedback: 54.6% rated it as useful [26], the average satisfaction score was 4.07 out of five [22], more than 80% reported it as helpful [23], and 91% of the users found the chatbot easy to use [20]. One study used user engagement indicators to identify high subjective usability (acceptability, usability, and satisfaction) but low objective engagement (completion, adherence, compliance, and engagement) [27]. Another study assessed chatbot performance metrics, reporting an intent detection accuracy of 99.1% and an entity extraction accuracy of 95.4% for the Mira Chatbot [18]. Chatbot usability was not evaluated in the remaining four studies [7,13,19,21].

**Table 2.** AI^a^ chatbot characteristics.

Author (year)/ Country	Chatbot name	Purposes of chatbot	AI chatbot type	AI algorithm	Mode of delivery	Interaction type	Initiates conversation	Completion related	Chatbot usability evaluation
Anmella et al [27] (2023)/Spain	Vickybot	To screen anxiety, depression, and burnout with interventions	Digital decision support platform (PRESTO^b^)	Machine-learning severity prediction model	Mobile app	1:1 interaction	Active	27% completed second self-assessment	High subjective UEI^c^ (acceptability, usability, and satisfaction). Low objective UEI (completion, adherence, compliance, and engagement)
Chaudhry and Islam [26] (2021)/United States	iChatBot (AI O) PeerChat (AI X)	To provide stress management support during COVID-19	Intelligent chatbot	iChatBot: machine learning model PeerChat: immediate communication with expert peers	Mobile app	iChatBot: 1:1 PeerChat: group chat	Passive (on-demand queries)	Not reported	54.6% participants rated the app as either useful or very useful 59.1% participants were willing to use it
Chang et al [22] (2024)/Singapore	Wysa	To evaluate Wysa for health professionals’ support	Conversational chatbot	Rule-based AI model	Mobile app	1:1 interaction with optional human coach support	Passive	Not reported	High engagement rate with positive feedback (91~93.2%) Mean feedback score of 4.07 (SD 0.95) out of 5
Jackson-Triche et al [23]/United States	UCSF^d^ Cope	To provide behavioral health support via chatbot	An algorithm-based, automated, web-based AI conversational tool	Natural language model	Web-based interface	1:1 interaction	Active	10.88% of employees accessed the chatbot	>80%of attendees reported the experience as helpful
Kleinau et al [20] (2024)/Malawi	Viki (Vitalk’s virtual mental health care assistant)	To assess chatbot impact on mental health in Malawi	Automated conversational agent	Rule-based AI model	Mobile app	1:1 interaction	Active	Of 1584 participants, 511 completed	91% found the app easy to use; 87% found the content relevant; 92% benefited from the app, and 83% felt more resilient Common barriers: lack of time (45%), app duration (30%), and usability issues (13%)
Kroon [21] (2018)/Netherlands	iThrive	To help health professionals manage inner critics and enhance self-compassion	Preprogrammed conversations and interaction	Natural language model	Web-based interface	1:1 interaction	Passive (users initiate interaction with the chatbot)	12 of 14 participants included in the final analysis	N/A^e^
Matsumoto et al [19] (2021)/Japan	CB^f^ app (a chatbot app)	To support mental health self-care for busy professionals, especially nurses	Digital decision support platform	Natural language model	Mobile app, VR^g^	1:1 interaction	Active	Not reported	N/A
Noble et al [7](2022)/Canada	MIRA^h^	To provide tailored mental health information and services for health professionals and their families	Hybrid NLP^i^ and decision tree AI chatbot	Natural language model	Web-based platform	1:1 interaction	Active	Not reported	N/A
Yoon et al [13](2021)/Singapore	mHealth^j^ app	To support frontline health professionals’s psychosocial well-being during COVID-19	N/A	Natural language model	Mobile app	1:1 interaction	Active	Not reported	N/A
Zamani [18] (2022)/Canada	MIRA	To offer strategic mental health resources for health professionals and their families	Hybrid NLP and decision tree AI chatbot	Natural language model	Web-based platform	1:1 interaction	Active	Not reported	Intent detection: 99.1% accuracy in identifying user needs Entity extraction: 95.4% accuracy in recognizing key terms for resource recommendations.

a AI: artificial intelligence.

b PRESTO: primary care digital support tool in mental health.

c UEI: user engagement indicator.

d UCSF cope: University of California, San Francisco faculty, staff, and trainee coping and resiliency program.

e Not available.

f CB: cognitive behavioral.

g VR: virtual reality

h MIRA: mental health intelligent information resource assistant.

i NLP: natural language processing.

j mHealth: mobile health.

### AI Chatbot Intervention Characteristics and Outcomes

Table 3 lists the contents and outcomes of the AI chatbot interventions. The primary component is the screening function for mental health conditions such as anxiety, depression, and stress, which was included in seven studies [19-23,26,27]. Four studies included cognitive behavioral therapy techniques [13,20,22,27]. Additionally, four studies implemented stress management techniques, such as breathing exercises and meditation [13,19,20,22]. Three studies incorporated problem-solving strategies [7,18,22] and psychoeducation [19,22,27]. Two studies used behavioral activation [20,22], cognitive restructuring [21,22], and gratitude training [20,22]. One study featured storytelling [21]. In addition, there was a function to support portal sites connected to community resources in three studies [7,18,23].

**Table 3.** AI^a^ chatbot intervention characteristics and outcomes.

Author (year)/ country	Chatbot contents	Additional features	Intervention doses	Main outcomes
Anmella et al [27](2023)/Spain	Screening and monitoring for self-assessment tools Personalized psychological modules: CBT^b^, mindfulness, DBT^c^, and ACT^d^ Psychoeducation and coping strategies for stress management Chatbot-guided navigation	Emergency alert for suicidal thoughts Weekly objective reminders Audio recording option for reflections after self-assessments	1 month (self-assessments conducted at baseline and biweekly)	Anxiety (*t*_8_=1.000; *P*=.34) and Depressive (*t*_8_=0.40; *P*=.70) symptoms were not significant differences Work-related burnout was moderately reduced (z=–2.07, *P*=.04, *r*=0.32)
Chaudhry and Islam [26] (2021)/United States	iChatBot—provides real-time answers (self-monitoring and stress-tracking information related to patient care and workplace stress management) PeerChat—enables peer-to-peer support	AssignedTasks: records stress levels and task completion times MyNotifications: Sends reminders for health activities like sleep, exercise, and meals SmartMonitor: connects with devices to track vitals (eg, blood pressure, oxygen, pulse, and temperature) InfectionCheck: Manual COVID-19 symptom checker via chatbot	N/A^e^	Usefulness and willingness: 54.6% found the app useful; 59.1% willing to use it. Six feature feedback: MyNotifications: useful reminders; PeerChat: positive for peer support; AssignedTasks: good for tracking, but time-consuming; InfectionCheck: useful, potential redundancy; StressMonitor: very helpful for coping; iChatBot: concerns about emergency responsiveness
Chang et al [22](2024)/Singapore	Mental health assessments: anxiety—GAD-7^f^, depression— PHQ^g^-2 Intervention modules: mindfulness, sleep meditation, guided visualization, thought recording, behavioral activation, psychoeducation, breathing exercises, cognitive restructuring, acceptance, grounding, social support, problem-solving, habit building, gratitude training	Option to connect with a human coach Weekly objective reminders and notifications Gratitude challenge to boost engagement Audio recording for self-reflection	Varied among users (on average, users completed 10.9 sessions over 3.80 weeks)	495 completed at least one full session; 422 completed two or more sessions The interventions most used were for sleep and anxiety, with a strong repeat-use rate 46.2% reported symptoms of anxiety 15.2% reported symptoms of depression
Jackson-Triche et al [23] (2023)/United States	Mental health assessment screening: suicide or self-harm thoughts, substance use concerns, emotional distress, social withdrawal and work-related struggles, resource navigation Web-based self-care resources Behavioral health services: emergency support	Telehealth assessments for crisis evaluation Self-management website offering curated resources In-person navigator for personalized support Support groups with regular peer meetings	Varied based on user interaction	UCSF^h^ cope reached all units 10.88% (3785/34,790) of employees accessed the technology 39.7% (708/1783) of employees with psychological distress requested in-person services Positive feedback on all program elements. Over 80% of attendees found the experience helpful
Kleinau et al [20](2024)/Malawi	Mental health assessments: anxiety (GAD-7), depression (PHQ-9), burnout (OLBI^i^), loneliness (UCLA Loneliness Scale)^j^, resilience (RS-14)^k^ Careline programs (thematic mental health conversation tracks) Intervention: CBT techniques, positive psychology strategies, behavioral activation, gratitude exercises, breathing, relaxation, and meditation exercises,	Mood tracking with emojis for human-like interaction Gamification for engagement Emergency support with local psychologist contact info for high-risk users	8 weeks -First 4 weeks: daily interactions Second 4 weeks: interactions every other day	GAD-7 showed a decrease (–0.44), but the result was not statistically significant. OLBI showed a decrease (–0.58), with borderline significance. PHQ-9 showed a significant decrease. UCLA loneliness scale: no significant RS-14 showed a significant increase.
Kroon [21](2018)/Netherlands	Awareness of inner critics: storytelling, encouraging reflection Identification of inner critics: assessment tool Cognitive restructuring techniques Self-compassion development	Humor: adds humor for engaging interactions Tokens: rewards for task completion Compliments: encourages users with motivating feedback	2 weeks	ProQOL^l^ and SCS^m^ no significant. Quantitative results: no significant changes in self-compassion or compassion fatigue. Qualitative results: Positive feedback on the iThrive intervention, with increased awareness of inner critics and changes in thinking patterns. Chatbot interactions reportedly reduced stress and improved self-compassion.
Matsumoto et al [19](2021)/Japan	Digital-SAT^n^ method: self-guided stress management, techniques to convert stress-related physical discomfort into positive sensations, image-based therapeutic exercises Mental health assessments: self-esteem, anxiety, emotional support, depression, problem-solving abilities Stress management: guided stress relief sessions, emotional stabilization exercises, visualization techniques	Automated daily messages with reminders and psychoeducational content Gamification elements such as progress tracking Immersive VR^o^ experiences	4 weeks	Self-esteem and family support significantly improved. Self-repression showed a trend of improvement but was not significant. SDS scores and counseling needs increased in the VR + CB^p^ group. Maximal blood pressure decreased significantly in the VR group and showed a decreasing trend in the VR + CB group. Minimal blood pressure significantly decreased in the VR group.
Noble et al [7](2022)/Canada	Mental health education and information: problem-solving Personalized resource navigation Self-help strategies and coping skills	Emergency contacts for urgent mental health support Hybrid AI approach: free-text input and predefined options Crisis support referrals for suicidal thoughts	N/A	The findings will be reported in follow-up research
Yoon et al [13](2021)/Singapore	Personalized goal setting: tailored goals for lifestyle improvements (eg, sleep, exercise), feedback on progress to encourage behavioral changes, reminders to support adherence to personal goals Educational resources: mindfulness exercises and short wellness studies, stress management techniques	In-app counseling and peer support Reminders and notifications Gamification elements: point-based rewards Engaging features to promote continued app use Mood tracking and progress monitoring	N/A	Mood-tracking reminders were helpful, but workers struggled with self-awareness. Goal-setting and resources were valued; frequent notifications were distracting. A built-in counselor chat was preferred for accessibility and privacy. Gamification was not well-received. Peer support was needed, but app-based interactions raised concerns. Motivation, usability, and rewards were crucial for app use.
Zamani [18](2022)/Canada	Information on mental health topics, including substance use and coping strategies: problem-solving Delivers personalized recommendations Facilitates access to local mental health services and programs through a resource portal	Emergency contact option for users experiencing distress Stores conversation logs Feedback system	N/A	Intent detection: 99.1% accuracy in identifying user needs. Entity extraction: 95.4% accuracy in recognizing key terms for resource recommendations.

a AI: artificial intelligence.

b CBT: cognitive behavior therapy.

c DBT: dialectical behavioral therapy.

d ACT: acceptance and commitment therapy.

e N/A: not available.

f GAD-7: general anxiety disorder-7.

g PHQ: patient health questionnaire.

h UCSF cope: University of California, San Francisco faculty, staff, and trainee coping and resiliency program

i OLBI: Oldenburg burnout inventory.

j UCLA: University of California, Los Angeles loneliness scale.

k RS-14: 14-item resilience scale.

l ProQOL: Professional Quality of Life Scale.

m SCS: Self-Compassion Scale

n SAT: structured association technique.

o VR: virtual reality.

p VR-CB: virtual reality-chatbot.

Across the studies, a variety of additional features were incorporated. Seven studies included AI chatbot functionalities for crisis management and support [7,13,18,20,22,27]. Specific features encompassed emergency contact information for crises [7,18,20,27], peer support groups and in-app forums for social support [13], expert support through in-app counseling, and options for connecting with human coaches [13,22,23]. Six studies incorporated reminders to encourage chatbot use [13,18,19,22,26,27]. Gamification elements, such as point-based rewards to encourage participation, were featured in four studies [13,19-21]. Peer support functionalities were available in three studies [13,23,26]. Two studies included mood-tracking features for health and lifestyle management [13,20], while another two studies provided an audio recording option [22,27]. Additionally, one study integrated devices for tracking vital signs [26] and provided an immersive virtual reality experience [19]. A total of 4 out of 10 studies explicitly stated the duration of chatbot use or intervention dose ([19]: 4 wk; [20]: 8 wk; [21]: 2 wk; [23]: 1 mo) while it was not specified in six studies. The dose varied among users in two studies [22,23].

The research in four studies evaluated psychological health outcomes [19-21,27], whereas, in six studies, the focus was primarily on the development and description of user perceptions of the AI chatbot without providing specific outcome measures [7,13,18,22,23,26]. The most commonly measured psychological health outcomes were anxiety and depression, which were examined in three studies [19,20,27]. Burnout was investigated in two studies [20,27]. Compassion fatigue [21], loneliness [20], resilience [20], and self-compassion [21] were also evaluated. Anxiety was measured in three studies: one reported a significant reduction of scores [20] while the other two reported no significant differences in anxiety scores [19,27]. Burnout was assessed in two studies, and each reported a significant decrease in its scores [20,27]. Depressive symptoms were measured in two studies: one reported a significant reduction of scores [20] while the other did not [27]. Other psychological variables were measured in one study each; of those, the variables that showed significant change in scores were health counseling needs [19] and resilience [20].

## Discussion

### Principal Results

The studies retrieved for this scoping review were published during the past seven years, between 2018 and 2024, and reflect the rapidly evolving trend of AI chatbot technology for supporting psychological health. The global distribution of studies across countries indicates a widespread interest in using AI for psychological health support and health professionals’ psychological health.

Our review revealed that chatbots were used by a broad spectrum of health professionals, highlighting their diverse applications. This diversity underscores the potential for universally designed chatbots to effectively address psychological health needs across different professional settings. However, prior research has shown that nurses, who have direct and continuous patient contact, experience greater mental health distress compared to other health professionals [24]. Therefore, future studies should consider incorporating role-specific options into chatbot designs to create tailored interventions for diverse professional groups.

The diverse research methods used in the retrieved studies signify the nascent stage of research on AI chatbots for psychological health support, emphasizing technology refinement and user experience comprehension. Such a variety of research approaches is crucial for validating the efficacy and safety of AI chatbot technology, enabling iterative improvements and a deeper understanding of how health professionals interact with AI chatbots [25]. However, it is noteworthy that only 4 out of 10 studies measured psychological health outcomes. This limitation underscores the significant gap between technological developments and clinical validation. As the field matures, it becomes imperative to quantitatively assess the impact of AI chatbots on psychological health indicators. Further studies measuring outcome variables to demonstrate the clinical effectiveness of AI chatbots are needed.

The studies reviewed here demonstrate the variety of AI algorithms used in chatbot design, including NLP-based-, rule-, and learning-based approaches. NLP-based AI chatbots which provide personalized responses by analyzing user inputs [28] are the most commonly used AI algorithms. Evidence has shown that an NLP-based AI chatbot, which simulates natural conversations, is successful at delivering tailored interventions [29]. Rule-based AI algorithms, adopted in 2 studies out of the 10 retrieved studies, are effective in providing fixed and predetermined responses; however, they lack the adaptability required to provide more dynamic and engaging psychological interventions [30]. Previous research has shown that NLP technology, which interprets psychological health cues from user-generated texts, significantly improves the effectiveness of interventions [31,32], a finding consistent with our review [7,13,18,19,21,23]. Moreover, the development of deep learning techniques has led to the widespread use of general-purpose pretrained language models in various NLP applications [33]. This suggests that NLP-based algorithms are particularly suitable for delivering personalized psychological support in real-time interactions. Machine learning and deep learning algorithms show promise in enhancing AI chatbots’ effectiveness by continuously learning through user interactions. These algorithms can provide accurate and personalized interventions over time [34]. Future studies should investigate how these advanced algorithms can be used to offer more precise psychological support tailored to individual user experiences.

User engagement is a critical factor in the success of mental health interventions; however, several studies did not report intervention completion rates [20,21,23,27]. These issues might become more pronounced in real-world settings, where factors such as interface complexity, the chatbot’s ability to provide meaningful and personalized feedback, and technical issues impact the overall user satisfaction and perceived effectiveness of the intervention [35]. This underscores the need for further exploration of how chatbot interactions can be designed to maintain user engagement, possibly through improved user experiences and personalization techniques [36,37]. From a different perspective, unlike traditional interventions, which are typically designed to be completed from start to finish, chatbot interactions often do not follow this linear structure. Users may terminate interactions once their immediate concerns are addressed, reflecting the unique flexibility of chatbots [38]. For instance, if a chatbot provides critical information for crisis management and the user responds affirmatively, the user is likely to exit the chat after their need is met. This immediate resolution highlights the efficiency of chatbot interventions in addressing user concerns promptly, even if the interaction appears incomplete. Recognizing this distinction is essential for accurately interpreting dropout rates and evaluating chatbot effectiveness.

The AI chatbots reviewed here comprised various therapeutic approaches. The incorporation of established psychological treatment techniques such as cognitive behavioral therapy, mindfulness, and dialectical behavior therapy into AI chatbots represents a novel approach to delivering psychological health interventions. This approach is considered novel because it uses AI to deliver evidence-based psychological techniques 24/7, serve multiple users simultaneously, tailor interventions based on individual responses, and combine various therapeutic modalities on a single platform.

This multidisciplinary therapeutic approach has the potential to address health professionals’ diverse psychological needs [31]. AI chatbot interventions are characterized by a digital and responsive design, which allows for integrating practical tools such as stress management techniques, mood tracking, and personalized goal setting [39]. These features align with the immediate and personalized support often required by health professionals. However, while personalization is a key advantage of AI chatbots, a need also exists for some level of standardization to ensure the interventions’ quality and effectiveness [40]. This standardization should focus on establishing evidence-based core components and assessment methods while still allowing for personalized delivery [31]. Future research should focus on identifying the most effective components of these chatbots and tailoring them to the specific psychological health needs of health professionals while maintaining a balance between standardization and personalization.

In this study, a temporary increase in depression scores was observed in one study [20], whereas previous research has reported AI chatbots’ positive effects in reducing anxiety, depression, and work-related burnout among health professionals [41]. These contrasting findings may reflect the challenges faced by such professionals, who frequently experience high levels of stress and emotional demands [42]. This suggests the complexity of psychological interventions and highlights the importance of careful monitoring to determine the appropriate duration and intensity of chatbot use.

Some studies in our review demonstrated the potential of AI chatbots to act as first-line responders in identifying and managing mental health crises. Prior research has also shown the potential benefits of AI chatbots not only in improving mental and emotional well-being but also in promoting behavioral changes [43]. While these features may only benefit a subset of users, their impact on preventing escalation to severe mental health conditions or emergencies is invaluable. Future research should prioritize the integration and evaluation of these features to enhance the safety and efficacy of chatbot interventions.

Although AI chatbots address the unique time constraints and stress among health professionals, balancing automated responses with genuine therapeutic interactions remains a significant challenge. Personalization technologies that provide tailored features to AI chatbots have emerged as key factors in their effectiveness, with studies reporting increased user engagement and satisfaction through customized responses and immediate support [29]. Consequently, the development of tailored intervention strategies that consider the individual needs and preferences of health professionals is essential to maximize AI chatbots’ effectiveness. These strategies should integrate ongoing user feedback and dynamically adapt interventions to maintain long-term user engagement and effectiveness. Future research should focus on larger sample sizes, extended follow-up periods, and diverse health care professional groups of health professionals. Such comprehensive studies will play a crucial role in developing evidence-based guidelines for implementing AI chatbots in psychological health and in advancing the sophistication of personalization algorithms.

### Limitations

This scoping review has several limitations. First, the limited number of studies adopting diverse study designs and using various AI algorithms complicated making direct comparisons among the retrieved studies. Second, the findings’ generalizability is restricted because many studies were published in specific regions, particularly high-income countries. Additionally, the short duration of many interventions and limited follow-up periods make it difficult to evaluate the long-term effectiveness and sustainability of AI chatbots. Future research should address these limitations by conducting more rigorous trials, standardizing the outcome measures, and exploring the long-term applications of AI chatbots across diverse populations and settings.

### Conclusions

This scoping review explored the current state of AI chatbots aimed at supporting the psychological health of health professionals. Although the reviewed studies demonstrated AI chatbots’ potential to reduce stress, anxiety, depression, and burnout, research in this area remains in its early stages. The diversity of study design, AI algorithms, therapeutic approaches, and outcome measures highlights this field’s innovative but fragmented nature.

Despite promising results, particularly with NLP-based chatbots, a significant need exists for more rigorous and standardized studies to fully evaluate their clinical efficacy. Challenges such as usability issues and limited generalizability of the findings must be addressed to enhance the long-term application and effectiveness of AI chatbots in real-world settings. Future research should focus on refining chatbot designs, expanding the research to diverse populations, and conducting long-term studies to clarify the role of AI chatbots in supporting the psychological health of health professionals. Thus, AI chatbots could become a valuable solution for addressing the psychological health challenges faced by health professionals worldwide.

## Supplementary material

10.2196/67682 Checklist 1 PRISMA-ScR checklist. The checklist has been completed in accordance with the journal's guidelines and includes all the items required for a systematic scoping review.

## Abbreviations

AI: artificial intelligence
NLP: natural language processing
